# Supplementary material for: Grouting mechanism and experimental study of goaf considering filtration effect
Source: PLoS One. 2023 Feb 24;18(2):e0282190. doi: 10.1371/journal.pone.0282190 (PMC9955605; doi:10.1371/journal.pone.0282190)
Supplement: Attachment — Submitted filename: Response to Reviewers.doc [file pone.0282190.s002.doc]

**Response to Reviewers’ Comments:**

We are really thankful to you for your marvellous efforts in our manuscript and give us constructive and meaningful comments, which can overwhelmingly improve the quality of our paper. Therefore, we have carefully revised the manuscript word by word. Thanks again for your work on our manuscript. The revised contents are marked in red fonts in the present manuscript.

**ACADEMIC EDITOR:**

**I carefully read through your manuscript. I feel that its content is enough to be acceptable for publication, but its writing-up should be significantly improved before any acceptance for publication. My comments are as follows:**

**1 Organization: Your focus is to determine the range of injected gangue slurry through its fluidity analysis. The content includes theoretical analysis and indoor experiment. What is their linkage between the two parts? The theoretical results should be compared with your experimental results.**

Response: Thank you for your advice. The theoretical analysis qualitatively shows that the water-cement ratio of slurry increases with the increase of diffusion distance under the effect of infiltration. In the laboratory test, by measuring the mass fraction of gangue particles at different locations after grouting, it was found that the mass fraction of coarse gangue particles at the location far from the grouting pipe was significantly reduced. This means that the water-cement ratio of the slurry at the position far away from the grouting pipe increases, which verifies the correctness of the theoretical analysis. Relevant contents have been added in sections 2.2 and 4.2 of the paper for the supplementary explanation.

**2 Theoretical analysis: This part is really confusing for reading because the formulae are not presented in a logical way. The principles or citations for the formulae development should be given.**

Response: Thank you for your advice. The theoretical part of section 2.2 of the paper was reorganized and written, and some important formulas were added to ensure the logic and correctness of theoretical derivation.

**3 Application to a project: Is this application example used to check your experimental observation or your theory? It should be analyzed or explained to serve your focus of this manuscript.**

Response: Thank you for your advice. According to the permeability gradient obtained from the test and the height of the grouting pipe, the grouting diffusion distance is predicted to be 51m. When the cumulative grouting volume of a single hole is 700 m3, the grout diffusion distance is expected to be 51 m according to the gravity flow gradient of the grout in the goaf of 6.34%. The accuracy of slurry seepage slope in goaf obtained from laboratory test is verified.

**4 English and presentations: Manuscript has many unclear statements or presentations. The novelty is not clearly presented. The citations in the text and references should follow the Journal style. Full revision or check should be done.**

Response: Thank you for your advice. The file has been modified according to the PLOS ONE style template and named as required. We have thoroughly checked and revised the manuscript's language usage, spelling and grammar.

**5 Conclusions: The items should be supported by data in your manuscript and enhanced to general knowledge. They will be useful for engineering practice or the understanding of injection flow mechanisms.**

Response: Thank you for your advice. According to your suggestion, I have refined the conclusion again and revised it as follows:

(1) Through theoretical analysis, the "water-cement ratio change matrix" in the coal gangue slurry seepage process is deduced to reveal the basic mechanism of the porous media filtration effect, and the water-cement ratio gradually increases in the seepage process of gangue slurry.

(2) The overall flow law of the gangue slurry in the goaf shows the law of first longitudinal expansion and then horizontal diffusion. Before the slurry is stabilized, the seepage gradient is large, and after stabilization, the seepage gradient decreases, the average seepage gradient is 6.34%.

(3) Filtration effect has an obvious space-time effect. The proportion of gangue particles less than 200 mesh in the gangue slurry near the grouting mouth is 9.81%, while the far end position is 20.36%. In the direction parallel to the infiltration direction, the water-cement ratio gradually increases with the diffusion distance. The farther away from the grouting mouth, the greater the water-cement ratio of the slurry.

(4) According to the permeability gradient obtained from the test and the height of the grouting pipe, the grouting diffusion distance is predicted to be 51m. The engineering application results show that the cumulative grouting volume of a single hole is 700 m^3^, the slurry diffusion distance is greater than 45 m, and the slurry has excellent fluidity in the void space of the goaf.

I am very grateful to the editorial department and the reviewers for their advice, which allowed me to carefully revise the manuscript, reorganize the paper, modify the keywords, title, and content, and take this opportunity to improve the academic quality and readability of the manuscript. Thanks again to the editorial department and the reviewers for their work. Your comments are all valuable and very helpful for revising and improving our paper.

Dr. GU

Xi'an City, Shaanxi Province, China

February 8, 2023
